# Application prospects of the 2BS cell-adapted China fixed rabies virus vaccine strain 2aG4-B40

**DOI:** 10.1186/s12985-024-02416-9

**Published:** 2024-07-08

**Authors:** Ying Xu, Lin Weng, Xuan Wang, Ming Li, Wanping Guo, Yiqing Liu, Xiang Li, Zhenping Wang, Xinyu Liu, Shengnan Xu, Feide He, Qianqian Hou, Tengzhou Li, Wenke Du, Yabo Zhang, Shumin Chang, Liwen Zhang, Yuntao Zhang

**Affiliations:** Beijing Institute of Biological Products Co., Ltd, Beijing, 100176 China

**Keywords:** Rabies virus, Vaccine, aG strain, 2BS, Adaptation

## Abstract

**Background:**

Rabies is a fatal zoonotic disease whose pathogenesis has not been fully elucidated, and vaccination is the only effective method for protecting against rabies virus infection. Most inactivated vaccines are produced using Vero cells, which are African green monkey kidney cells, to achieve large-scale production. However, there is a potential carcinogenic risk due to nonhuman DNA contamination. Thus, replacing Vero cells with human diploid cells may be a safer strategy. In this study, we developed a novel 2BS cell-adapted rabies virus strain and analysed its sequence, virulence and immunogenicity to determine its application potential as a human diploid cell inactivated vaccine.

**Methods and results:**

The 2BS cell-adapted rabies virus strain 2aG4-B40 was established by passage for 40 generations and selection of plaques in 2BS cells. RNA sequence analysis revealed that mutations in 2BS cell-adapted strains were not located at key sites that regulate the production of neutralizing antibodies or virulence in the aG strain (GQ412744.1). The gradual increase in virulence (remaining above 7.0 logLD50/ml from the 40th to 55th generation) and antigen further indicated that these mutations may increase the affinity of the adapted strains for human diploid cells. Identification tests revealed that the 2BS cell-adapted virus strain was neutralized by anti-rabies serum, with a neutralization index of 19,952. PrEP and PEP vaccination and the NIH test further indicated that the vaccine prepared with the 2aG4-B40 strain had high neutralizing antibody levels (2.24 to 46.67 IU/ml), immunogenicity (protection index 270) and potency (average 11.6 IU/ml).

**Conclusions:**

In this study, a 2BS cell-adapted strain of the 2aG4 rabies virus was obtained by passage for 40 generations. The results of sequencing analysis and titre determination of the adapted strain showed that the mutations in the adaptive process are not located at key sequence regions of the virus, and these mutations may enhance the affinity of the adapted strain for human diploid cells. Moreover, vaccines made from the adapted strain 2aG4-B40 had high potency and immunogenicity and could be an ideal candidate rabies virus strain for inactivated vaccine preparation.

**Supplementary Information:**

The online version contains supplementary material available at 10.1186/s12985-024-02416-9.

## Introduction

Rabies is a lethal zoonotic disease caused by rabies virus infection in the central nervous system [1]. Currently, the rabies virus still poses a major threat to human health worldwide, especially in the developing countries of Asia and Africa. It causes approximately 59,000 human deaths per year [2]. Rabies virus is part of the family Rhabdoviridae and genus Lyssavirus. The rabies virus genome is a single-stranded negative-sense RNA genome consisting of 11928 or 11932 nucleotides. From the 3’ end to the 5’ end of the genome, five genes (N, P, M, G and L) encode the following proteins: nucleoprotein (N), phosphoprotein (P), matrix protein (M), glycoprotein (G), and RNA-dependent RNA polymerase (L) [3]. In the RNA genome, the N, P, and L proteins form a ribonucleoprotein complex that is responsible for virus replication. The M protein surrounds the nucleocapsid, forming a bridge between the NC and the viral envelope [4, 5]. The G protein, the only viral protein exposed on the surface, is the main factor that determines the pathogenicity of the rabies virus and is the only antigen that is responsible for stimulating the body to produce neutralizing antibodies [6–9]. After the rabies virus enters the periphery postexposure, the virus attaches to cellular receptors, enters host cells by fusion with the cellular membrane and subsequently spreads into the central nervous system, causing neuronal dysfunction and leading to a fatal outcome [10–13]. The average incubation period after infection is 1–3 months, but it has been documented to last from several weeks to more than a year [14]. The main clinical symptoms of rabies include fever, paralysis, delirium, convulsions and hydrophobia. Once clinical manifestations are present, the patient typically dies within 10 days.

Rabies is one of the oldest infectious diseases known in human history and has been prevalent for more than 4,000 years [15]. There are no effective treatments for rabies. In 1885, Louis Pasteur developed the first rabies virus vaccine from the spinal cord of rabies virus-infected rabbits. Since the first development of the rabies vaccine by Pasteur, the efficacy of strategies to prevent rabies in humans has increased. Due to the extremely high mortality of rabies, only inactivated rabies vaccines have been approved for human rabies control. Immunization with inactivated rabies vaccines preexposure or within 24 h postexposure combined with/without the administration of rabies immunoglobulin can be an effective strategy against the disease [16]. Currently, there are many types of vaccines on the market around the world. Purified duck/chick embryo cell vaccines were developed and proven to have suitable immunogenicity. However, due to the limited cell division ability of primary cultured cells, large-scale vaccine production is difficult [17–19]. Moreover, people with egg allergies cannot use these vaccines. The Vero cell rabies vaccine [20] can be mass-produced and has suitable immunogenicity, but there are some potential risks, such as DNA contamination and tumorigenicity. A human diploid cell-adapted rabies vaccine for human use was produced by adapting the fixed virus strain to human diploid cells, such as the foetal lung MRC-5 cell line. Human diploid cell vaccines also have suitable immunogenicity and are safer than other rabies vaccines [21]. However, lower virus yields and higher production costs make it difficult to scale up the production of vaccines [22, 23]. Thus, developing human diploid cell-adapted strains that are more suitable for large-scale vaccine production is highly important for rabies prevention.

The aG strain is derived from the Beijing strain, which is a rabies virus isolated from the brain of a rabies-infected dog that was then injected into the brain of a rabbit to make it a fixed strain. After 31 passages, it can be used to prepare rabies vaccines. In 1965, the Beijing strain of rabies virus was alternately adapted to in guinea pig brain cells and primary gopher kidney cells for 15 years, after which an adaptive strain of gopher kidney cells was obtained, which was named the “aG strain”. The amino acid homology between the aG strains and the isolated original virus was approximately 87.2-92.9%. To date, the aG strain has been inactivated through multiple passages in cells and used as a vaccine strain in China. This study aimed to develop a human diploid cell-adapted rabies virus strain that can be used to provide safe, effective, highly consistent human antigenicity and high-yield rabies vaccines. In our study, the aG-fixed virus was adapted to guinea pig brain cells to generate the 2aG4 strain, and the 2aG4 strain was adapted to 2BS cells by the combination of passaging and picking plaques. Then, we performed sequencing, mass spectrometry, titre analysis and identification tests for generations of the virus strains obtained by adaptive passage. Finally, culture, harvest, concentration, purification, inactivation and lyophilization of a vaccine with suitable protective agents were performed using the adapted strain, and the immunogenicity and potency of the vaccine were tested. Our results imply that the vaccine serves as an effective prophylactic agent for rabies infection.

## Methods

### Animals

All animal procedures were approved by the Institutional Animal Care and Use Committee of Beijing Institute of Biological Products Co., Ltd. Male mice aged 3–4 weeks were used for the experiments.

### 2BS cell culture

The human diploid cell line 2BS was derived from the lung tissue of an aborted 3-month-old foetus and was established in 1974. The cell line has a long life cycle and can be passaged for 66 generations. 2BS cells were cultured in DMEM supplemented with 10% foetal bovine serum (FBS) (HyClone, USA) for 6–8 days. Then, the cells were digested and passaged. The passaged cells were used for rabies virus culture.

### Western blot analysis

Proteins were extracted from the rabies virus samples using ice-cold RIPA lysis buffer. The total protein concentrations of the samples were measured by a BCA assay (Pierce, USA). Proteins were then separated on SDS‒PAGE gels and dyed with Coomassie brilliant blue or transferred to PVDF membranes (Merck Millipore, Germany). The membranes were blocked with 3% w/v bovine serum albumin for 1 h and then incubated with primary antibodies against the rabies virus G protein (HyTest 3R7-4F1, China) overnight at 4 °C. After washing with 1× TBST, the membranes were incubated with the appropriate secondary horseradish peroxidase-conjugated goat anti-rabbit IgG antibody (Biodragon, China). Immunoblots were visualized with enhanced chemiluminescence (ECL) reagent and evaluated using a ChemiDoc XRS + instrument (Bio-Rad, Hercules, CA, USA).

### Coomassie bright blue staining

The SDS‒PAGE gel with proteins was put into a protein staining apparatus (eStain LI, Genscript). The gel was stained for 2 min with Coomassie blue for one cycle and eluted for 2.5 min with decolorizing fluid for two cycles.

### Sequencing

Nucleotide sequencing was performed by allwegene Beijing (Beijing, China). Genomic RNA was extracted from the viruses at different passage numbers, and the nucleic acid concentration was determined by a Qubit instrument. A VAHTSTM Universal DNA Library Prep Kit for Illumina V3 was used for library construction. After the library was sequenced with a NextSeq550 instrument (Illumina, USA), clean reads were obtained after quality control, bwa software (v0.7.17-r1198-dirty) was used to conduct in-depth comparison analysis with the reference genome (aG strain (GQ412744.1)), and the comparison rate and coverage were calculated.

### Enzyme-linked immunosorbent assay (ELISA)

We assessed antigen levels by ELISA. Anti-aG strain antibodies were extracted from mouse sera and used to coat the ELISA plate. The anti-aG strain antibody was added to the 96-well plate and incubated at 37 °C for 1–2 h, followed by incubation at 4 °C overnight. The next day, the 96-well plates were blocked with 3% BSA for 1 h at 37 °C. Subsequently, the cells were added and cultured for 1 h. After incubation with the enzyme-labelled antibody for 30 min at 37 °C, TMB one solution and termination solution were added sequentially. The absorbance (OD value) of each well was measured by a microplate reader at wavelengths of 450 nm and 630 nm, and the final OD value was determined by subtracting the 630 nm absorbance value from the 450 nm absorbance value.

#### Virus plaque formation

2BS cells were seeded on six-well plates, and the rabies virus adapted to the 2BS cells was added at the same time. Then, 2BS cells and the rabies virus were cocultured in an incubator at 37 °C with 5% CO2 overnight. The next day, the culture solution was discarded, 0.5% methylcellulose was added, and 2BS cells infected with rabies virus were cultured for another 6 days. When cytopathic or suspected lesions could be seen on the six-well plates, the lesion site was selected. The selected plaques were placed in 96-well plates with a 2BS cell suspension and cultured in an incubator at 37 °C with 5% CO2 for 6 days. The supernatant was then harvested and transferred to 24-well plates with a 2BS cell suspension. After culturing for 6 days, the supernatant was harvested, and the antigen content was detected. The viruses with high antigen content were selected for further culture with 2BS cells. The virus titre was detected after 3 generations of the rabies virus with high antigen content adapted on 2BS cells, and the virus with the highest titre was selected for further culture.

#### Mass spectrometry

Proteins were extracted from rabies virus samples, and the protein structures were disrupted by treatment with the surfactant RapigestTM SF (Waters Corporation, USA). Then, the disulfide bond was opened by DTT (EMD Millipore Corporation, USA), followed by blocking with IAM (EMD Millipore Corporation, USA). The proteins were finally digested with trypsin, and the reaction was terminated by formic acid. Peptide separation was carried out on an Acquity Pertide BeyC18 column (Waters Corporation, USA), and the samples were collected by a Thermo Scientific Q Exactive Plus mass spectrometer. The collected data were analysed with BioPharma Finder software. Samples with low bias (Delta ≤ 5 ppm), a confidence score of 100, and the detection of both parent and daughter ions were compared.

### Direct fluorescent antibody test

A direct fluorescent antibody test was used to identify the rabies antigens of 2aG4-B40. BHK-21 cells were plated on 96-well plates and allowed to form a monolayer. Then, the cells were infected with the virus (diluted 5-fold series) for 24 h at 37 °C. Next, the cells were fixed with 80% ice-cold acetone and stained with isothiocyanate FITC-labelled N-protein. The plates were observed via fluorescence microscopy.

### Virus titration

The virus titre was measured via intracerebral injection in mice as reported in the Chinese Pharmacopoeia. Briefly, the brains of NIH mice in different groups (6 mice for each group) weighing 11 ~ 13 g were inoculated with 0.03 ml of serial 10-fold dilutions of virus, and nonspecific deaths that occurred within three days after inoculation were excluded. The numbers of mice that developed clinical symptoms and died were scored after 14 days. The median lethal dose (LD50) and virus titres were calculated using the Reed and Muench method.

### Rabies virus identification

The specific properties of the 2aG4-B40 strain were identified by an intracerebral neutralization test in mice. The virus was diluted 10-fold in series, and the appropriate dilutions of virus were mixed with rabies immunoglobulin (neutralization group) or negative serum (control group) in equal amounts. Groups of NIH mice (6 mice in each group) weighing 11 ~ 13 g were inoculated intracerebrally with 0.03 ml of the mixture at each dilution, and nonspecific death within three days after inoculation was excluded. The numbers of mice that developed clinical symptoms and died were scored after 14 days. The median lethal dose (LD50) and virus titres were calculated using the Reed and Muench method. The neutralization index is the antithesis of the control virus titre minus the neutralization virus titre.

### Preparation of the test vaccine

The harvested 2aG4-B40 strains were first concentrated on 300 kDa ultrafiltration membranes (Millipore Pellicon 2, USA) and then purified on chromatographic columns. The purified virus was inactivated with β-propanolactone. The test vaccine was then formulated with the purified virus at an antigen concentration of 200 mIU/mL. The test vaccine was lyophilized with suitable protective agents in a vacuum freeze-drier (Shanghai Kyowa Vaccum Engineering Co., Ltd.) through a lyophilization process consisting of three stages: freezing at -40 °C, primary drying at -3 °C and secondary drying at 28 °C.

### Immunogenicity studies

Preexposure prophylaxis (PrEP) and postexposure prophylaxis (PEP) vaccination are effective means to prevent rabies disease. Thus, the immunogenicity of the test vaccines was determined by PrEP and PEP vaccination.

The PrEP vaccination regimen was administered by intraperitoneal injection on D0 and D7. Specifically, NIH mice (12–14 g) were injected with 0.5 ml of test vaccines on Day 0 and Day 7. The negative control mice were injected with 0.5 ml of PBS in the same way. Both groups of mice were administered the standardized virus (CVS strain, 0.03 ml, 10 mice per dose) in serial 10-fold dilutions by intracerebral injection on Day 14. The numbers of mice that developed clinical symptoms and died were scored after 14 days. The median lethal dose (LD50) was calculated using the Reed and Muench method. The protection index was determined as the ratio of the LD50 between the vaccine group and the control group.

As recommended by the WHO, the five-dose regimen (Essen, 1-1-1-1-1) was one of the PEP IM regimens. To determine the rabies virus neutralizing antibody concentration (RVNA) in mice, the rapid fluorescent focus inhibition test (RFFIT) [24] was used to evaluate the serum samples that were collected at Days 0, 7, 14, 28 and 42.

### Vaccine potency test

The NIH test (scheme) was selected as the detection method to evaluate the protective efficacy of the adapted vaccine against infection in mice and compare it to that of the national standard rabies vaccine. Different dilutions of the national standard and test vaccines were intraperitoneally injected into NIH mice (12–14 g, 0.5 ml for each mouse, 10 mice per dose) on Day 0 and Day 7. Then, all of the mice were challenged with standardized virus (CVS strain, 0.03 ml) via intracerebral injection on Day 14. Titration of the CVS strain was indispensable for this test. The median effective dose (ED50) was calculated using the Reed and Muench method. The relative potency of the test vaccines were obtained by comparison with the ED50 of the national standard.

### Statistical analyses

Statistical data are presented as the mean ± SEM (standard error of the mean). GraphPad Prism version 9.0 was used for all the statistical analyses. Paired two-tailed Student’s *t* tests were used to compare two groups, and one-way analysis of variance (ANOVA) was used to compare differences among > 2 groups. All of the statistical experiments were repeated at least three times independently. *P* < 0.05 was considered to indicate statistical significance.

## Results

### Process of adapating the 2aG4 strain to 2BS cells

**Fig. 1:**
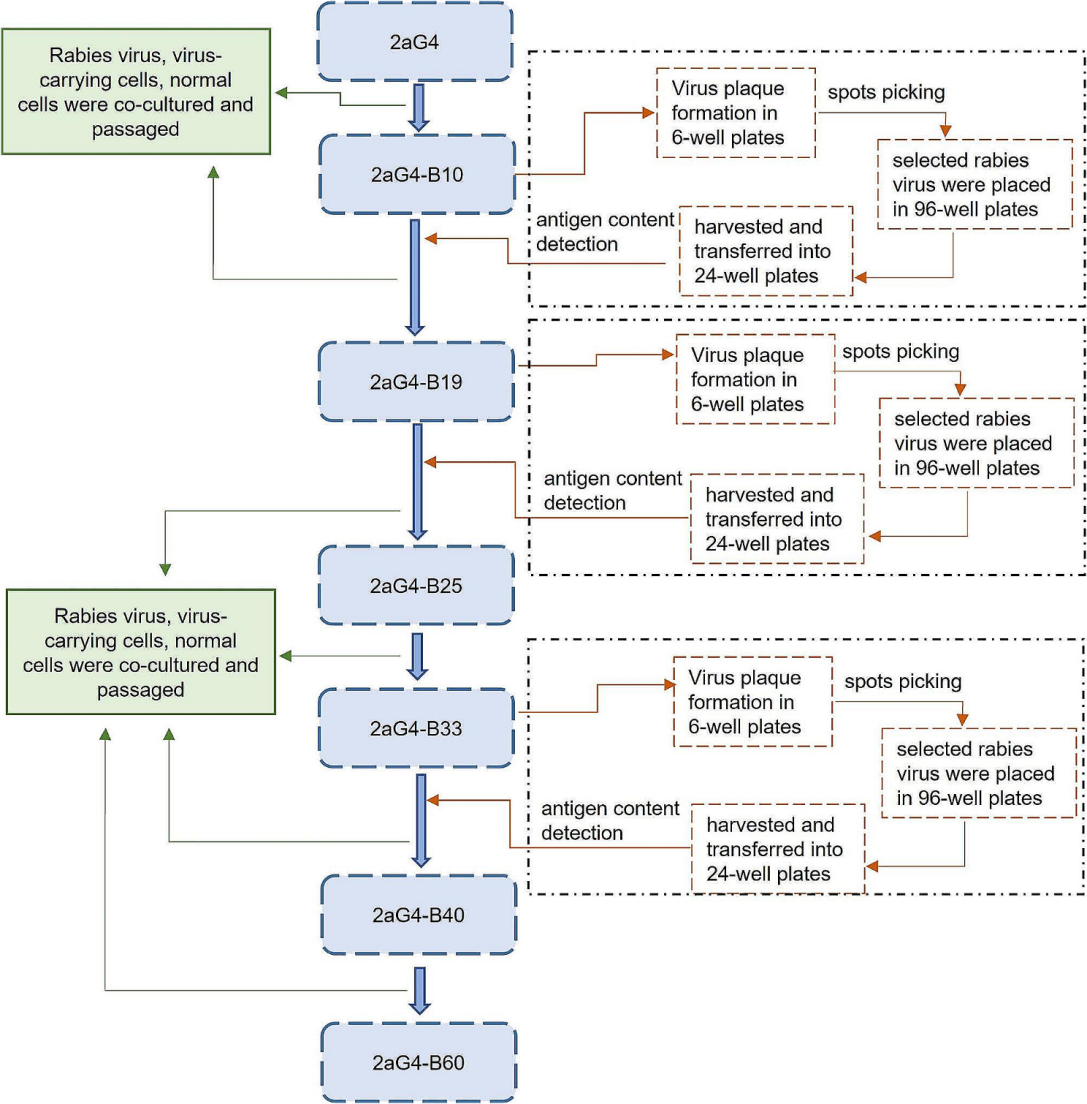
Adaptive passage of the 2aG4 strain in 2BS cells. The fixed rabies virus aG strain (2aG4) was adapted to human diploid cells by the combination of passaging and selecting plaques in 2BS cells, and the virus was selected and cloned by selecting plaques at the 10th, 19th, and 33rd generations

First, the fixed rabies virus aG strain (2aG4) was adapted to human diploid cells by the combination of passaging and selecting plaques from 2BS cells (Fig. 1). When the virus titre reached a certain level, a 10-fold series of diluted virus supernatant and 2BS cells were cocultured in a 6-well plate for plaque picking, and viruses with high antigen content were selected and cloned. The human diploid cell-adapted rabies virus strain was obtained after several passages of continuous adaptation.

The fixed rabies virus 2aG4 strain (donated by Wuhan Institute of Biological Products) was cocultured with 2BS cells, and the supernatant was harvested after 6 days of cultivation and recorded as 2aG4-B1 (B1 indicates that the virus was adapted to 2BS cells for one generation). Then, the virus-carrying cells were passaged at a ratio of 1:2 and mixed with appropriate non-virus-carrying 2BS cells, and 2aG4-B1 virus supernatant was added for culture at the same time. Subsequently, the virus obtained was recorded as 2aG4-B2 (B2 indicates that the virus was adapted to 2BS cells for two generations, and so on in a similar fashion). 2BS cells carrying the 2aG4 strain were passaged to the 10th generation; subsequently, the 10th generation viruses were selected and cloned by plaque selection. Virus selection and cloning took place on passage 10, 19, and 33, and the virus titre tended to increase. After each plaque selection, the virus titre decreased; however, with increasing passage time, the virus titre increased progressively. Finally, the 40th-generation virus strain 2aG4-B40 was able to undergo stable subculture and was established as a human diploid cell-adapted strain of the rabies virus.

### Sequence comparison of the genomes of the 2BS cell-adapted rabies virus strain 2aG4-B and strain aG

**Fig. 2:**
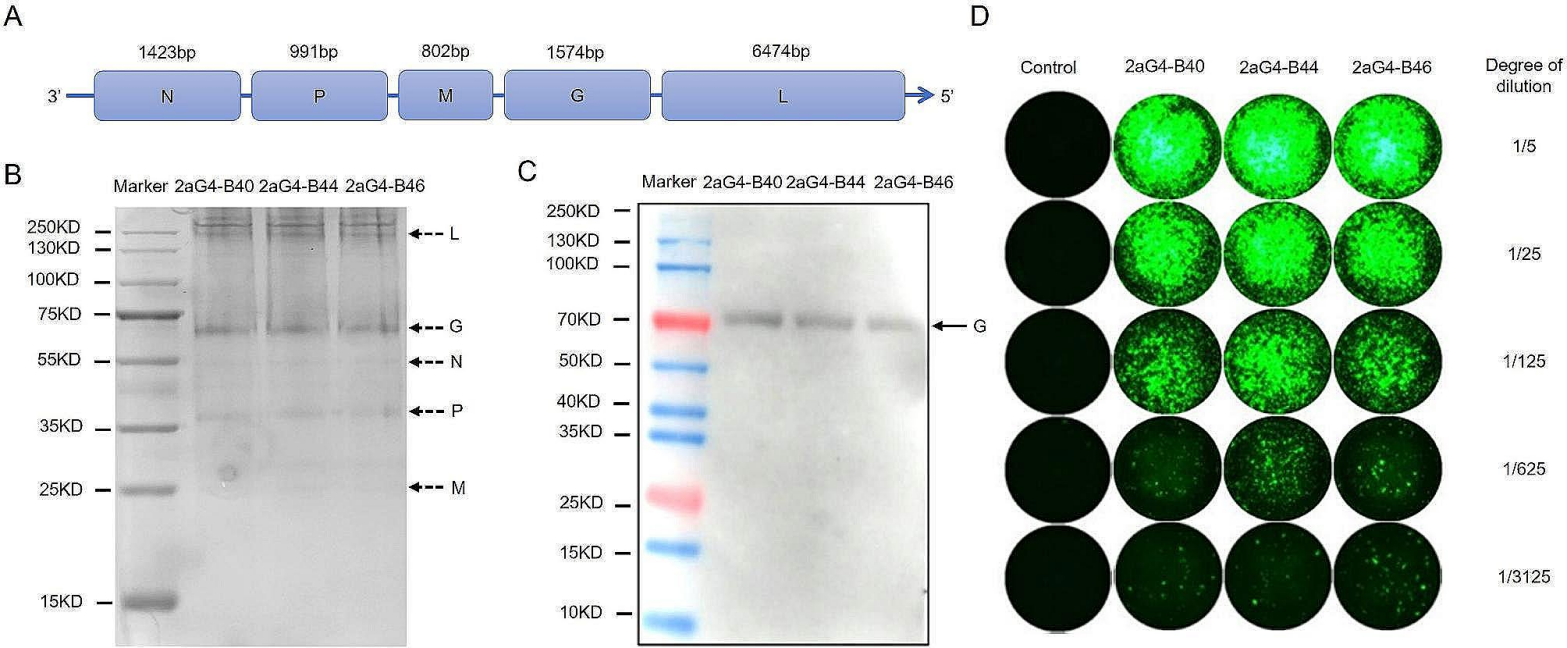
Rabies virus proteins expression in the 2BS cell-adapted rabies virus strain. (**A**) The rabies virus genome encoding the N, P, M, G and L proteins from the 3’ to 5’ ends. (**B**) Coomassie bright blue staining showing protein expression in the 2BS cell-adapted rabies virus strains 2aG4-B40, 2aG4-B44 and 2aG4-B46. *n* = 1. (**C**) Representative Western blot showing G protein expression in the 2BS cell-adapted rabies virus strains 2aG4-B40, 2aG4-B44 and 2aG4-B46. *n* = 1. (**D**) Immunofluorescence showing N protein expression in 2BS cells infected with the 2BS cell-adapted rabies virus strains 2aG4-B40, 2aG4-B44 and 2aG4-B46. *n* = 1

The rabies virus genome encodes nucleoprotein (N), phosphoprotein (P), matrix protein (M), glycoprotein (G) and polymerase (L) from the 3’ to 5’ ends (Fig. 2A). SDS‒PAGE and mass spectrometry revealed that the adapted strain contained rabies virus proteins, and the molecular weights of these proteins in 2aG4-B40 were 23 kDa (M protein), 42 kDa (P protein), 50.5 kDa (N protein), 72 kDa (G protein) and 244 kDa (L protein) (Fig. 2B; Table 1). Western blot results also showed that the human diploid cell-adapted rabies virus contained G protein (Fig. 2C). The direct N protein fluorescent antibody test of the 2BS cell-adapted strain (passages 40, 44 and 46) showed that there was an apparent green fluorescence intensity in all groups with a virus dilution of 1/5, and the number of green fluorescent foci clearly decreased with increasing virus dilution (Fig. 2D), indicating that N protein expression was increased in the 2BS cell-adapted strain.

**Table 1:** Mass spectrometry of 2BS adapted rabies virus 2aG4-B40

Group	MS area	MW (KD)
Glycoprotein	2640049.14	72
Matrix protein	1501123.69	23
Nucleoprotein	951765.85	50.5
Phosphoprotein	821839.69	42

To investigate the sequence variation during the adaptation process of 2aG4 to 2BS, the genome sequence of the 2BS cell-adapted virus was measured, and the homology and protein mutations of the sequence compared with those of the aG strain (GQ412744.1) were analysed by SnapGene software (Tables 2 and 3). For 2aG4-B10 (10th generation), 2aG4-B19 (19th generation) and 2aG4-B31 (31st generation), the G protein sequences were determined; for 2aG4, 2aG4-B40 (40th generation), 2aG4-B44 (44th generation), 2aG4-B46 (46th generation), 2aG4-B48 (48th generation), 2aG4-B50 (50th generation), 2aG4-B55 (55th generation) and 2aG4-B60 (60th generation), the full length of RNA sequences was determined. The results showed that the homology of the full-length RNA sequences between the 2BS cell-adapted strain and the reference aG strain was greater than 99.41% (2aG4 and 40–60 generations), and the homology of the G protein sequence was greater than 99.68% (0–60 generations) (Table 2). Five base mutations occurred in the G protein, causing four amino acid mutations; seven base mutations occurred in the P protein, causing five amino acid mutations; one base mutation occurred in the M protein, causing one amino acid mutation; and two base mutations occurred in the N protein, causing no amino acid mutations (Table 3). All of these mutations occurred before the 40th generation, and the 2BS cell-adapted rabies virus genome remained stable from 40 to 60 passages in 2BS cells, except for the mutation in the M protein, which could be traced back to the original strain. The next step is to test whether the mutations had effects on the titre, potency and immunogenicity of the 2BS cell-adapted rabies virus strain and the resulting vaccine.

**Table 2:** Homology comparison of 2BS adapted rabies virus and the aG strain

Rabies virus	Full-length sequence homology	Number of mutation sites of G protein gene	G protein sequence homology
aG	/	/	/
2aG4	99.52%	1	99.87%
2aG4-B10*	/	3	99.87%
2aG4-B19*	/	4	99.81%
2aG4-B31*	/	4	99.81%
2aG4-B40	99.41%	5	99.68%
2aG4-B44	99.42%	5	99.68%
2aG4-B46	99.42%	5	99.68%
2aG4-B48	99.42%	5	99.68%
2aG4-B50	99.43%	5	99.68%
2aG4-B55	99.43%	5	99.68%
2aG4-B60	99.43%	5	99.68%

* Represents the length of the G protein sequence

**Table 3:** Sequence comparison of 2BS adapted rabies virus and the aG strain Sequence

Proteins	*N*		*P*							M	G				
Genome positions	1333	1414	1743	1748	1878	2135	2219	2275	2351	2969	4419	4427	4611	4688	4822
Amino acid sites	421	448	77	78	122	207	235	254	279	158	368	371	432	458	502
aG	C/Ile	C/Ser	C/His	T/His	G/Val	G/Glu	C/Asp	G/Gly	A/Glu	G/ Ser	G/Gly	C/His	A/Glu	G/Glu	G/Val
2aG4	A/Ile	C/Ser	C/His	A/Gln	G/Val	A/Glu	T/Asp	A/Glu	A/Glu	G/ Ser	G/Gly	C/His	A/Glu	G/Glu	C/Val
2aG4-B10	/	/	/	/	/	/	/	/	/	/	A/Glu	T/Tyr	A/Glu	G/Glu	C/Val
2aG4-B19	/	/	/	/	/	/	/	/	/	/	A/Glu	T/Tyr	G/Gly	G/Glu	C/Val
2aG4-B31	/	/	/	/	/	/	/	/	/	/	A/Glu	T/Tyr	G/Gly	G/Glu	C/Val
2aG4-B40	A/Ile	T/Ser	A/Asn	A/Gln	A/Met	A/Glu	T/Asp	A/Glu	C/Asp	A/Asn	A/Glu	T/Tyr	G/Gly	A/Lys	C/Val
2aG4-B44	A/Ile	T/Ser	A/Asn	A/Gln	A/Met	A/Glu	T/Asp	A/Glu	C/Asp	A/Asn	A/Glu	T/Tyr	G/Gly	A/Lys	C/Val
2aG4-B46	A/Ile	T/Ser	A/Asn	A/Gln	A/Met	A/Glu	T/Asp	A/Glu	C/Asp	A/Asn	A/Glu	T/Tyr	G/Gly	A/Lys	C/Val
2aG4-B48	A/Ile	T/Ser	A/Asn	A/Gln	A/Met	A/Glu	T/Asp	A/Glu	C/Asp	A/Asn	A/Glu	T/Tyr	G/Gly	A/Lys	C/Val
2aG4-B50	A/Ile	T/Ser	A/Asn	A/Gln	A/Met	A/Glu	T/Asp	A/Glu	C/Asp	G/ Ser	A/Glu	T/Tyr	G/Gly	A/Lys	C/Val
2aG4-B55	A/Ile	T/Ser	A/Asn	A/Gln	A/Met	A/Glu	T/Asp	A/Glu	C/Asp	G/ Ser	A/Glu	T/Tyr	G/Gly	A/Lys	C/Val
2aG4-B60	A/Ile	T/Ser	A/Asn	A/Gln	A/Met	A/Glu	T/Asp	A/Glu	C/Asp	G/ Ser	A/Glu	T/Tyr	G/Gly	A/Lys	C/Val

### Virulence and antigen changes in the adaptation process of the 2aG4-B strain

**Fig. 3:**
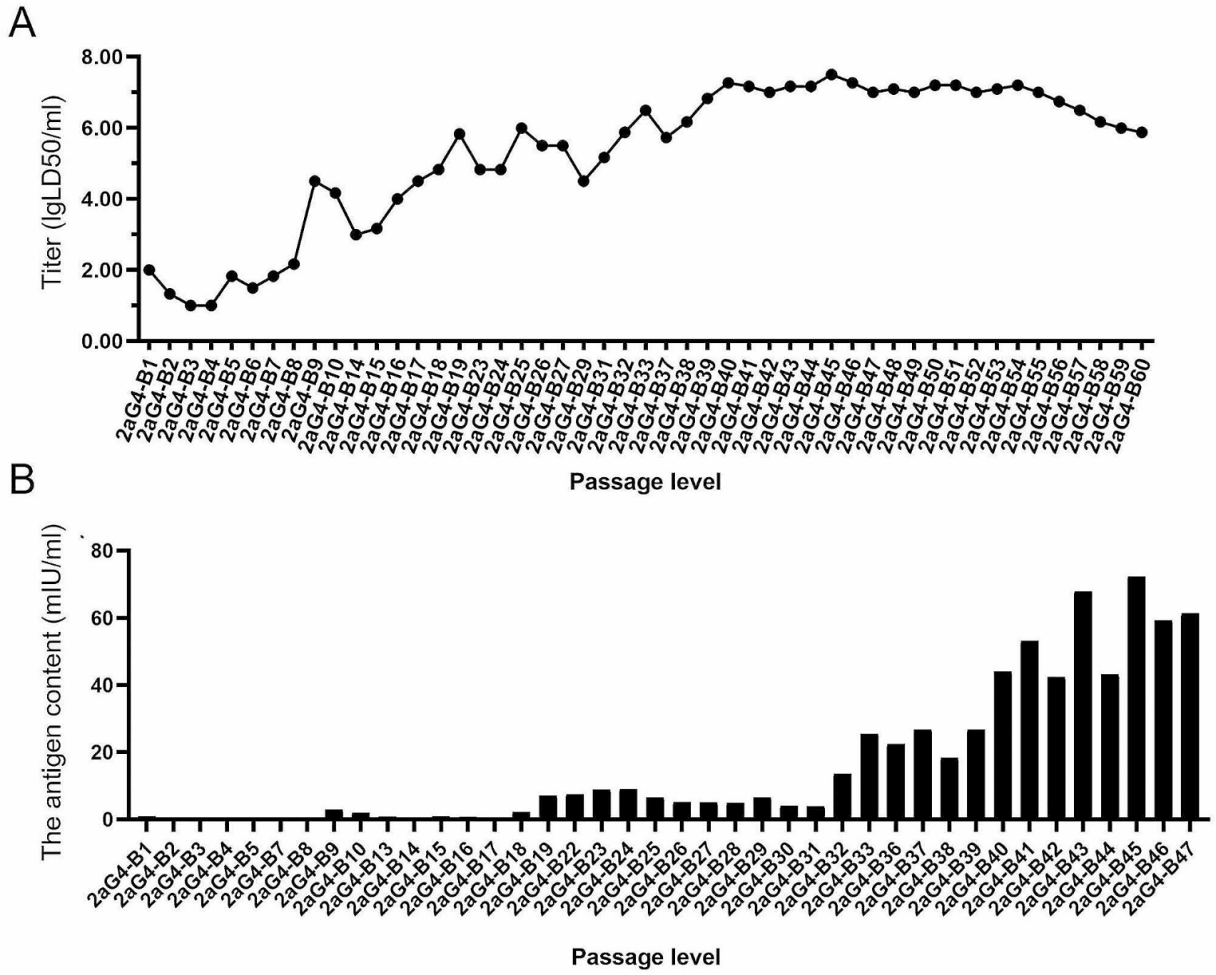
Virulence and antigen changes in the adaptation process of the 2aG4-B strain. (**A**) The intracerebral injection method showed changes in the virus titre after passage in 2BS cells from 2aG4-B1 to 2aG4-B60. *n* = 1. (**B**) ELISA showing the changes in virus antigen after passage in 2BS cells from 2aG4-B1 to 2aG4-B47. *n* = 1

The virus titres were monitored in mice to analyse the virus propagation properties during passage in 2BS cells (Fig. 3A). Only by serial passages in 2BS cells did the titre of the 2aG4 strain increase slowly despite the decreasing trend during the initial 10 passages to approximately 2.00-4.50 logLD50/ml. A combination of plaque screening at random and propagation in 2BS cells at 10, 19 and 35 passages were used to increase virus virulence. The titre of the virus increased to 7.27 logLD50/ml at the 40th generation (2aG4-B40) and remained above 7.0 logLD50/ml until the 55th generation (2aG4-B55). The antigen content also remained at a high level after the 40th generation (Fig. 3B). However, the virus titres gradually decreased after further passage in 2BS cells, reaching 5.88 logLD50/ml at passage 60. The above results suggested that the 2aG4 strain adapted to 2BS cells, and there were no significant differences in viral titre or RNA sequence among the 40th- to 55th-generation viruses; they were all defined as human diploid cell-adapted strains of rabies virus. Hence, the virus strain 2aG4-B40 was used for further study.

### Identification of the rabies virus

**Fig. 4:**
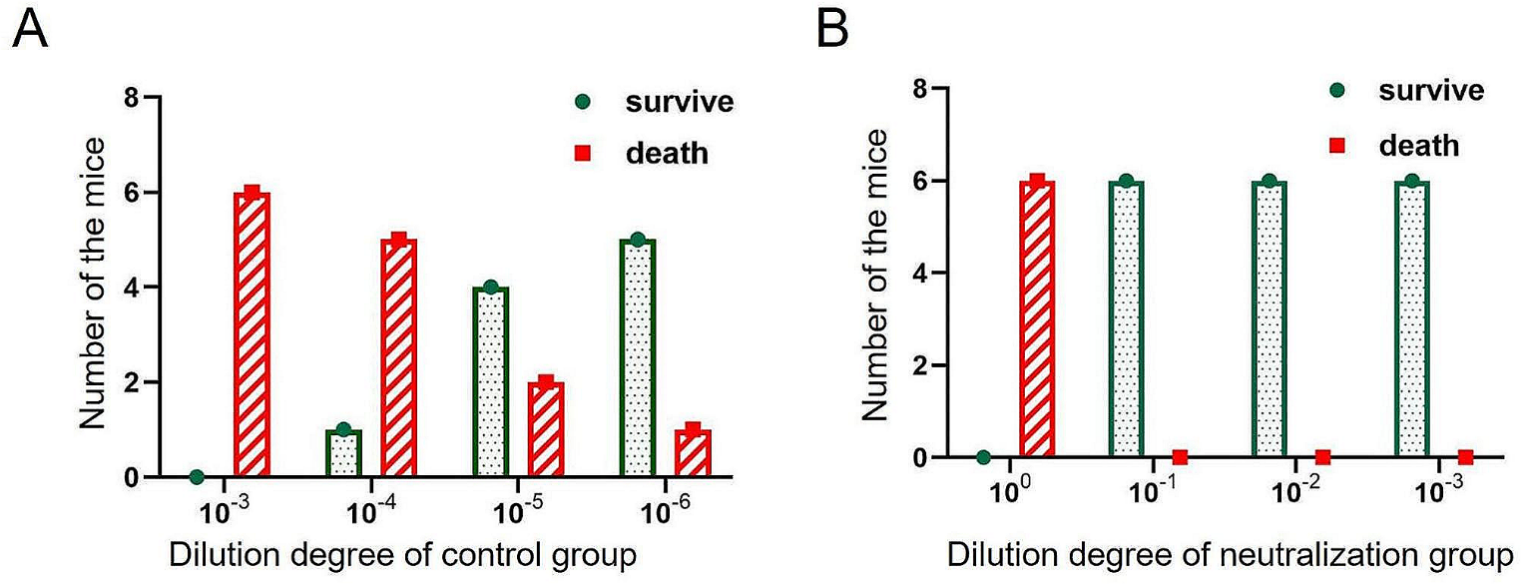
Identification of the rabies virus 2aG4-B40 strain. (**A-B**) The neutralization test showed the number of surviving and dead mice in the control group (**A**) and the neutralization group (**B**) on the 14th day after injection. *n* = 6 mice for each group

To further characterize the properties of the 2aG4-B40 strain, we performed an intracerebral neutralization test in mice. The number of surviving mice in the control group decreased with increasing virus concentration (Fig. 4A), and all the mice died at a virus dilution of 10^0^ in both the control and neutralization groups (Fig. 4A and B). However, after mixing rabies immunoglobulin with the virus (at dilutions ranging from 10^− 3^ to 10^− 1^), all the mice in the neutralization group survived (Fig. 4B). The results indicated that rabies immunoglobulin could be used to identify the 2aG4-B40 antigen. According to the Reed and Muench method, the logLD50/mL of the control group was 6.3, and that of the neutralization group was 2.0. Hence, the neutralization index of the 2aG4-B40 strain was 19,952, which was much greater than the WHO-recommended standard of 500.

### Immunogenicity and potency of the vaccine prepared with the 2aG4-B40 strain

**Fig. 5:**
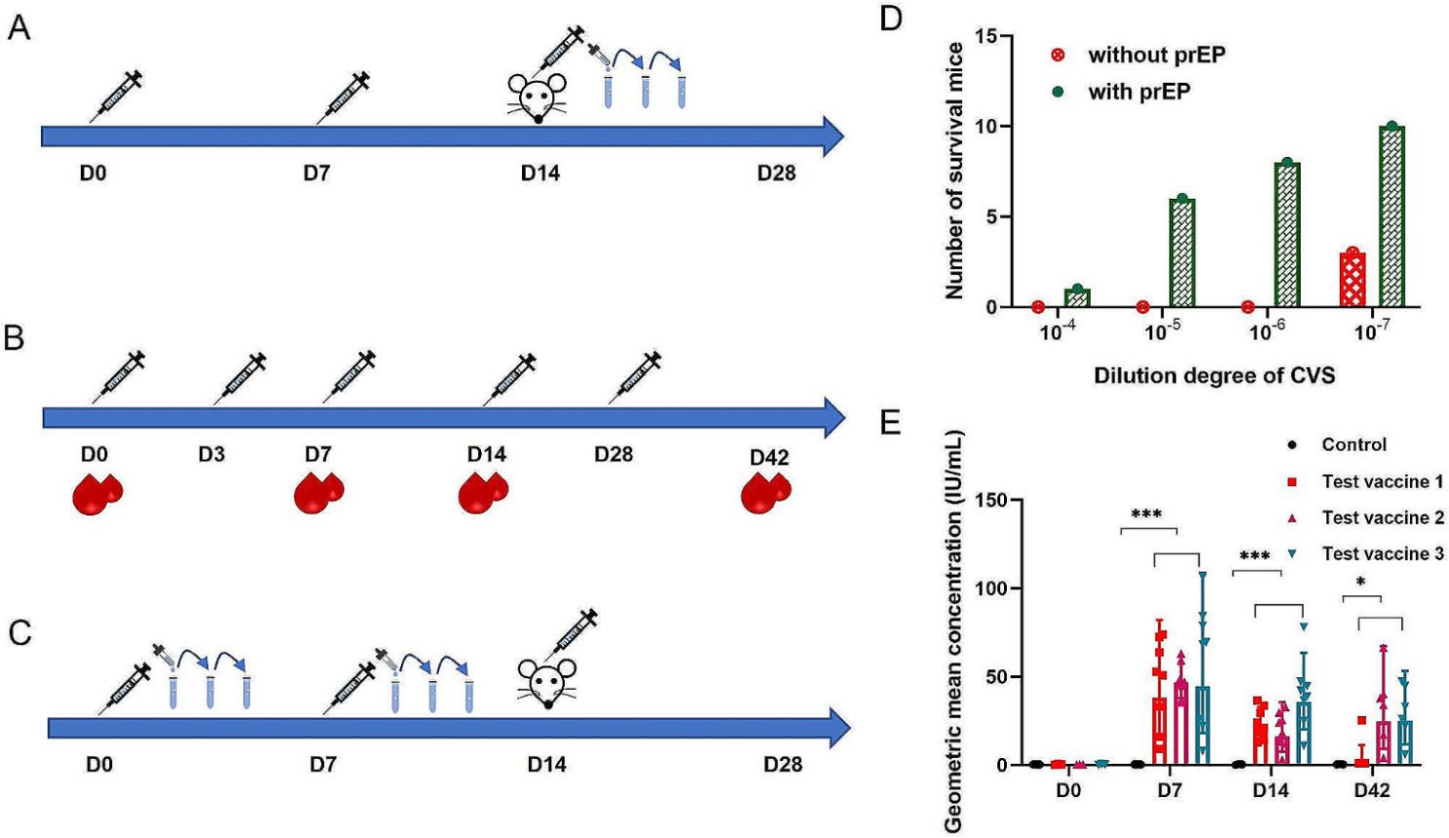
Immunogenicity and potency of the vaccine prepared with the 2aG4-B40 strain. (**A**) The 2-dose PrEP IM regimen for the test rabies vaccines. (**B**) The five-dose PEP IM regimen (Essen, 1-1-1-1-1) for the test rabies vaccines. (**C**) The 2-dose IM regimen of the NIH test for potency. (**D**) Immunogenicity test showing the number of surviving and dead mice in the test vaccine injection group and control group after challenge with the standardized CVS virus at different dilutions with the PrEP IM regimen. *n* = 10 mice for each group. (**E**) Immunogenicity test showing the anti-rabies antibody levels in mice immunized with test vaccines prepared from 2aG4-B40. *n* = 8 mice for each group. Statistical significance was assessed by a nonpaired 2-tailed Student’s t test

To further investigate whether the 2BS cell-adapted strain has the potential to be an ideal candidate rabies virus strain for inactivated human vaccine preparation, the strain was developed into a test vaccine. The test vaccines were prepared by culture, harvest, concentration, purification, inactivation and lyophilization with suitable protective agents using the 2aG4-B40 strain. First, the immunogenicity of the 2aG4-B40 strain was determined by using the PrEP IM regimen (Fig. 5A) to confirm the protection index. All mice died when challenged with the standardized CVS virus at dilutions ranging from 10^− 4^ to 10^− 6^ (Fig. 5D). However, the test vaccines obviously protected the mice from death induced by the standardized CVS virus, and the number of surviving mice increased with increasing dilutions of the standardized CVS virus (Fig. 5D). According to the Reed and Muench method, the logLD50/mL of standardized CVS virus in the PrEP group was 6.42, and that in the control group was 8.85. Hence, the protection index of 2aG4-B40 was 270, which met the WHO-recommended standard of 100.

According to the 5-dose PEP IM regimen (Fig. 5B), the anti-rabies antibody levels of the mice immunized with the test vaccines are shown in Fig. 5E. The test vaccines were formulated at a fixed antigen concentration of 200 mIU/ml with three batches of the harvested 2aG4-B40 strain virus. The mice produced high levels of neutralizing antibody (geomean concentration: 37.98 ~ 46.67 IU/ml), and the neutralizing antibody concentration reached a plateau at 7 days after the first immunization. After that, the antibodies decreased gradually to a relatively low level but remained above 0.5 IU/ml (geomean concentration: 2.24 ~ 24.99 IU/ml) within 42 days after the first PEP vaccination. In addition, the seroconversion rates were 100% in all groups from 7 days to 42 days after the first immunization (Table 4).

**Table 4:** The anti-rabies antibody seroconversion rates of mice immunized with test vaccines by 5-dose PEP IM regimen

Group	D0	D7	D14	D42
Test vaccine 1	0%	100%	100%	100%
Test vaccine 2	0%	100%	100%	100%
Test vaccine 3	0%	100%	100%	100%

Finally, the NIH test (Fig. 5C) was used to verify the potency of the test vaccine. The results showed that the ED50 of the national standard was 2.61 IU/ml, which was within the reasonable range of 2.10 to 2.75 IU/ml. Three independent experiments showed that the average potency of the test vaccines was approximately 11.6 IU/ml (Table 5), which was greater than the WHO-recommended standard of 2.5 IU/ml.

**Table 5:** The potency of test vaccines prepared from the 2aG4-B40 through NIH test

Group	ED50	Potency (IU/mL)
Test vaccine 1	2.58	10.7
Test vaccine 2	2.65	12.6
Test vaccine 3	2.61	11.6
National standard of China	2.61	11.4

## Discussion

In the present study, we developed a 2BS cell-adapted rabies virus strain by adapting the aG strain (2aG4) to human diploid cells (2BS cells) via the combination of passaging and plaque selection. The strain underwent several mutations during its adaptation process. The results of sequencing analysis and titre determination of the adapted strains indicated that the mutations increased the affinity of the adapted strains for human diploid cells during the adaptation process but did not decrease the titre or immunogenicity of the adapted strains. The vaccines prepared by culture, harvest, concentration, purification, inactivation and lyophilization with suitable protective agents using the 2aG4-B40 strain had high potency and increased immunogenicity, indicating that the adapted strain has the potential to be used for vaccine preparation.

Glycoprotein (G), which is composed of 524 amino acids, is the main factor determining the pathogenicity of the rabies virus and is the only antigen that stimulates the body to produce neutralizing antibodies [8]. The sequencing comparison results showed that the homology of the G protein sequence between the 2BS cell-adapted strain and the reference aG strain was greater than 99.68% (0–60 generations). The homology of the 2BS cell-adapted strain G protein sequence (40–60 generations) was approximately 89.40% compared to that of the CVS-11 strain, 84.79% compared to that of the CTN-1 strain, and 84.41% compared to that of the Chinese street strain CGX89-1 (data not shown). The G protein antigen region contains three neutralizing antigenic sites, namely, antigenic site II (amino acids 34–200), antigenic site III (amino acids 330–357), minor antigenic site I (amino acids 218–240) and several linear neutralizing antibody epitopes, such as G5 (amino acids 253–275). Amino acids at positions 34 ~ 42, 147, 184, 198 ~ 200, 333, 336 and 338 are key amino acids, and at positions 342 and 343, there may be a new antigen site [25]. The 333 amino acid Arg is associated with neural invasion and transsynaptic transmission ability and is considered to be the key residue involved in rabies virus neurotropism and virulence [26, 27]. Sequencing revealed that 5 mutations in the G gene of 2BS cell-adapted strains (0–60 generations) affected 4 amino acid residues in the G protein: G368E, H371Y, E432G and E458K. However, none of these mutations were located at the key amino acid site, suggesting that these mutations had no effect on the antigenicity of the rabies virus and did not affect the production of neutralizing antibodies.

Phosphoprotein (P) contains approximately 297 amino acids, and the 186–297 positions are the key amino acid residues that affect viral replication [28]. Sequencing analysis revealed that the H77N, H78Q, V122M, G254E, and E279D amino acid mutations in the P protein of the 2BS cell-adapted strains (40–60 generations) were located at key sites that affect virus replication. Although amino acid mutation sites 254 and 279 are located at the key sites of the P protein that regulate viral replication, the virulence results during passage in 2BS cells showed that the viral titre of 2BS cell-adapted strains (40–55 generations) remained above 7.0 logLD50/ml, suggesting that the mutation did not significantly affect viral replication ability. The matrix protein (M) contains approximately 202 amino acid residues and plays a regulatory role in the transcription and replication of the rabies virus [29], virus assembly, morphogenesis and budding. The M protein also influences pathogenicity by interacting with G proteins [30]. Two antigenic regions (amino acids 17–72 and 50–171) of the M protein have been identified [31] and may participate in the immune response of the virus. The sequencing results showed that compared with the aG strain, the M protein was mutated at position 158 in the 2aG4-B40, 2aG4-B44, 2aG4-B46 and 2aG4-B48 generations, from serine to asparagine. Although amino acid 158 is located in the antigenic region of the M protein, the M protein antigen does not affect the production of neutralizing antibodies. The N protein consists of 450 amino acids, and its phosphorylation promotes viral transcription and replication. Compared to the aG strain, although the N gene of 2BS cell-adapted strains (40–60 generations) possessed 2 nucleotide mutations at bases 1333 and 1414, neither of them affected the amino acid sequence. These results showed that the key epitopes of the rabies virus strains that stimulate the production of neutralizing antibodies did not change during the process of adapting to human diploid cells. Although mutations at the amino acid sites G254E and E279D in the P protein may affect viral replication, the high titre of 2BS cell-adapted strains (40–55 generations) indicated that the mutation did not significantly affect this factor. In addition, all of these mutations occurred before the 40th generation, and the 2BS cell-adapted rabies virus genome remained stable from 40 to 60 passages in 2BS cells.

Compared to the Vero cell rabies vaccine, the human diploid cell vaccine also has suitable immunogenicity but is safer. Despite the risk of DNA contamination and tumorigenicity, the Vero cell rabies vaccine is currently the most widely used vaccine due to its high production and moderate cost. Moreover, lower virus yields and higher production costs make it difficult to scale up human diploid cell vaccines. Therefore, it is highly important to develop a vaccine with high yield and improved safety. The adapted strains were demonstrated to multiply and grow in 2BS cells at high titres, and identification tests showed that the 2BS cell-adapted virus strain was neutralized by anti-rabies serum with a neutralization index of 19,952, which confirmed that the 2BS cell-adapted strain was indeed a rabies virus. The titre of the virus increased to 7.27 logLD50/ml at the 40th generation (2aG4-B40) and remained above 7.0 logLD50/ml until the 55th generation (2aG4-B55). Four base mutations occurred in the G protein, causing four amino acid mutations, and one base mutation occurred in the M protein, causing one amino acid mutation (Table 3) between the 40th generation (2aG4-B40) and the fixed rabies virus 2aG4 strain (2aG4-B0). The cytopathic effects were illustrated to clarify the affinity of 2aG4-B40 for 2BS cells. As shown in Figure S1, there were no obvious differences between normal cells and cells cultured with 2aG4-B0 after 7 days. However, at the same MOI, the cytopathic degree of cells cultured with 2aG4-B40 for 7 days significantly increased. According to the increased virus titres and significant cytopathic effects, we assumed that the mutations may increase the affinity of the adapted strains for human diploid cells. The 2aG4-B40 strain was prepared as a test vaccine by culture, harvest, concentration, purification, inactivation and lyophilization with suitable protective agents. The high levels of neutralizing antibody (2.24 to 46.67 IU/ml), immunogenicity (protection index 270) and potency (average 11.6) of the test vaccine indicated that the 2BS cell-adapted virus strain had suitable preventive and protective effects against rabies virus infection.

## Conclusion

In this study, we established a 2BS cell-adapted strain (from 2aG4-B40 to 2aG4-B60) by passing the 2aG4 strain into 2BS cells for serial passages. Sequencing analysis of the adapted strain showed that mutations during the adaptation process increased the affinity of the adapted strains for human diploid cells without affecting their immunogenicity or titre. Vaccines made from the adapted strain 2aG4-B40 had high levels of neutralizing antibodies, immunogenicity and potency, which makes it an ideal candidate rabies virus strain for inactivated human vaccine preparation.

### Electronic supplementary material

Below is the link to the electronic supplementary material.

Supplementary Material 1

## Data Availability

No datasets were generated or analysed during the current study.

## Abbreviations

N: Nucleoprotein
P: Phosphoprotein
M: Matrix protein
G: Glycoprotein
L: RNA-dependent RNA polymerase
Vero: Cells African green monkey kidney cells
PrEP: Preexposure prophylaxis
PEP: Postexposure prophylaxis
LD50: Median lethal dose
ED50: Median effective dose
CVS: Challenge virus standard
RFFIT: Rapid fluorescent focus inhibition test
