# Supplementary material for: Application prospects of the 2BS cell-adapted China fixed rabies virus vaccine strain 2aG4-B40
Source: Virol J. 2024 Jul 8;21:154. doi: 10.1186/s12985-024-02416-9 (PMC11229241; doi:10.1186/s12985-024-02416-9)
Supplement: Supplementary file 1 — Supplementary Material 1 [file 12985_2024_2416_MOESM1_ESM.docx]

**Supplementary materials**


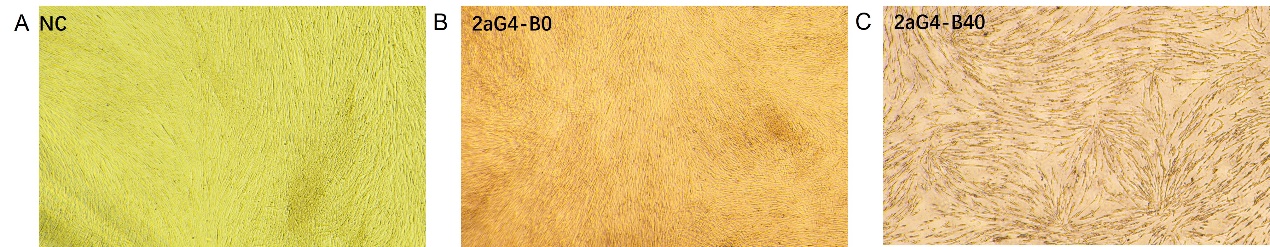


**Figure S1. Cytopathic effects in 2BS cells.** (A) Normal 2BS cells cultured for 7 days. (B) 2BS cells co-cultured with 2aG4-B0 for 7 days. (C) 2BS cells co-cultured with 2aG4-B40 for 7 days.
